# Nachhaltigkeitsrisiken in Versicherungsunternehmen. Regulatorische Entwicklungen, Szenarioanalysen und Stress-Tests

**DOI:** 10.1007/s12297-022-00521-8

**Published:** 2022-03-16

**Authors:** Mirko Kraft

**Affiliations:** Fakultät Wirtschaftswissenschaften, Hochschule für angewandte Wissenschaften Coburg, Friedrich-Streib-Str. 2, 96450 Coburg, Deutschland

## Abstract

In diesem Beitrag werden regulatorische Entwicklungen zu Nachhaltigkeitsrisiken in Versicherungsunternehmen betrachtet. Nachhaltigkeitsrisiken werden anhand der ESG-Kriterien differenziert (environmental, social, governance). Als weitere Differenzierung wird die in physische und transitorische Risiken gewählt. Nachgezeichnet werden die regulatorischen Entwicklungen auf globaler, europäischer und nationaler Ebene. Schwerpunkt liegt auf den Arbeiten zu EU-Vorgaben durch die Europäische Kommission und die EU-Versicherungsaufsichtsbehörde EIOPA, insbesondere zum EU-Aufsichtssystem Solvency II und zu Versicherungs-Stress-Tests. Der Fokus richtet sich auf die Entwicklung von Szenarioanalysen und Stress-Tests zu Nachhaltigkeitsrisiken. Letztere sind für mikro- und makroprudenzieller Zwecke möglich. Ihre Ausgestaltung wird u. a. hinsichtlich Szenarien, Zeithorizonten und Granularität diskutiert. Der Beitrag gibt somit einen Überblick und Ausblick auf die regulatorische Behandlung von Nachhaltigkeitsrisiken im Versicherungssektor, wobei die EU-Vorgaben Leitbild für internationale Fortschritte in diesem Feld sein könnten.

## Abstract (en)

This paper looks at regulatory developments on sustainability risks in insurance companies. Sustainability risks are differentiated based on ESG criteria (environmental, social, governance). A further differentiation is made between physical and transitory risks. Regulatory developments at global, European, and national level are traced. The focus is on the work on EU regulation by the European Commission and the EU insurance supervisory authority EIOPA, especially on the EU supervisory system Solvency II and on insurance stress testing exercises. The focus is on scenario analyses and stress tests on sustainability risks. The latter are applicable for micro- and macroprudential purposes. Their design is discussed with regard to, inter alia, scenarios, time horizons and granularity. The article thus provides an overview and outlook on the regulatory treatment of sustainability risks in the insurance sector, whereby the EU requirements could serve as a model for international progress in this field.

## Einleitung

Versicherer standen in der Vergangenheit nicht im Mittelpunkt der Debatten zum Klimawandel^1^ oder zu Nachhaltigkeit. Dies ist darauf zurückzuführen, dass das traditionelle Versicherungsgeschäft keine energieintensive Branche mit einem großen ökologischen Fußabdruck und hohen CO_2_-Emissionen ist. Die (direkte) Umweltverschmutzung durch Versicherungsunternehmen ist gering, sodass Versicherer nicht als die Verursacher der Klimakrise anzusehen sind. Die politische Diskussion im Zusammenhang mit dem sog. Pariser Klimaabkommen^2^ und den Nachhaltigkeitszielen der Vereinten Nationen^3^ hat jedoch auch die Finanzmärkte und insbesondere die Versicherungsbranche erfasst.

Nachhaltigkeit ist ein Projekt Europas, das durch den europäischen „Grünen Deal“ 2019 politisch auf EU-Ebene konkretisiert wurde.^4^ Er wurde von der (neuen) Europäischen Kommission (unter ihrer Präsidentin Ursula von der Leyen) vorgestellt und von den EU-Mitgliedstaaten angenommen. Europa will 2050 der erste klimaneutrale Kontinent sein.^5^

Das Pariser Klimaabkommen enthält die Neuausrichtung der Kapitalströme auf nachhaltige Investitionen, um ein nachhaltiges und integratives Wachstum zu erreichen. Darüber hinaus sollten relevante finanzielle Risiken, die sich aus dem Klimawandel ergeben, bewertet und gemanagt werden, ebenso wie die Erschöpfung von Ressourcen, Umweltzerstörung und soziale Fragen. Die Förderung von Transparenz und Langfristigkeit in der Finanz- und Wirtschaftstätigkeit ist ebenfalls Teil des Ziels.

Angesichts der zunehmenden Bedeutung dieses Themas hatte die Europäische Kommission im Mai 2018 bereits ein legislatives Maßnahmenpaket zu nachhaltigen Finanzen veröffentlicht. Ziel war es, Anlegern den Vergleich des CO_2_-Fußabdrucks ihrer Investitionen zu erleichtern, indem ein einheitliches EU-Klassifizierungssystem für nachhaltige Wirtschaftstätigkeiten, eine Taxonomie, geschaffen wird. Die anhaltenden Diskussionen dazu zeigen, dass eine Einteilung in „grün“ und „braun“ keineswegs trivial ist. Noch weniger einfach ist es, Nachhaltigkeitsrisiken in Versicherungsunternehmen und -gruppen tatsächlich zu identifizieren und zu bewerten. Insbesondere die Risikomessung von Nachhaltigkeitsrisiken stellt – gegenüber klassischen finanziellen Risiken – eine erhebliche Herausforderung dar. Mit Szenarioanalysen und Stress-Tests zu Nachhaltigkeitsrisiken kann dieser begegnet werden, wobei Fragen der unternehmensindividuellen, aber auch standardisierten Ausgestaltung von hoher Bedeutung sind.

Nach dieser Einführung (1.), die den Hintergrund der Nachhaltigkeitsdebatte erläutert, folgen Grundlagen der Nachhaltigkeit, z. B. die Differenzierung von Nachhaltigkeitsrisiken anhand von sog. ESG-Kriterien sowie der Differenzierung in physische und transitorische Risiken (2.). Dann wird ein Überblick über ausgewählte regulatorischen Entwicklungen im Bereich der nachhaltigen Finanzwirtschaft in Bezug auf Versicherungsunternehmen gegeben (3.). Unterschieden werden die globale, die europäische und die nationale Ebene. Im Kap. 4 werden mögliche Szenarioansätze zur Integration des Nachhaltigkeitsrisikos in den ORSA-Prozess von Versicherungsunternehmen unter Solvency II vorgestellt. Darüber hinaus wird in Kap. 5 die Ausgestaltung von Mikro-Stress-Tests bis hin zu Makro-Nachhaltigkeits‑/Klima-Stress-Tests an ausgewählten Referenz-Punkten diskutiert, u. a. dem Zeithorizont und den gewählten Annahmen. Der Beitrag endet mit einer Zusammenfassung und einem Ausblick (6.).

## Nachhaltigkeit

### Begriff Nachhaltigkeit und Nachhaltigkeitsrisiken

Der Begriff „Nachhaltigkeit“ (bzw. „sustainability“) wird vielfach verwendet. Eine einheitliche Definition ist in der Literatur und in der Praxis nicht zu erkennen. Umgangssprachlich wird häufig „nachhaltig“ (bzw. „sustainable“) als etwas angesehen, dass dauerhaft erscheint, wobei diese Beständigkeit positiv assoziiert wird. Vielfach wird in der Literatur auf historische Entwicklungen Rückgriff genommen, die z. B. zum einen auf den sprachlichen Ursprung des Begriffs „Nachhaltigkeit“ oder anderer naheliegender Begriffe wie beispielsweise „corporate social responsibility“ (CSR) eingehen^6^ und zum anderen auch auf eine globale Dimension des Bewusstseinswandels.^7^ Als dessen Ankerpunkt wird z. T. der sog. Brundtland-Bericht von 1987 ausgewählt.

In die letztere Richtung geht auch das Nachhaltigkeits-Verständnis der Europäischen Kommission im Kontext ihrer Strategie für eine nachhaltige Finanzwirtschaft („sustainable finance“):^8^

Es geht darum, dafür zu sorgen, dass künftige Generationen die gleichen oder bessere Möglichkeiten haben als wir, und gleichzeitig die begrenzten Ressourcen unseres Planeten zu wahren.

Es erscheint nicht sinnvoll, in diesem Beitrag die begrifflichen Definitionen um eine neue zu erweitern oder die verschiedenen Definitionsansätze vertiefend zu diskutieren.

**Figure 1:**
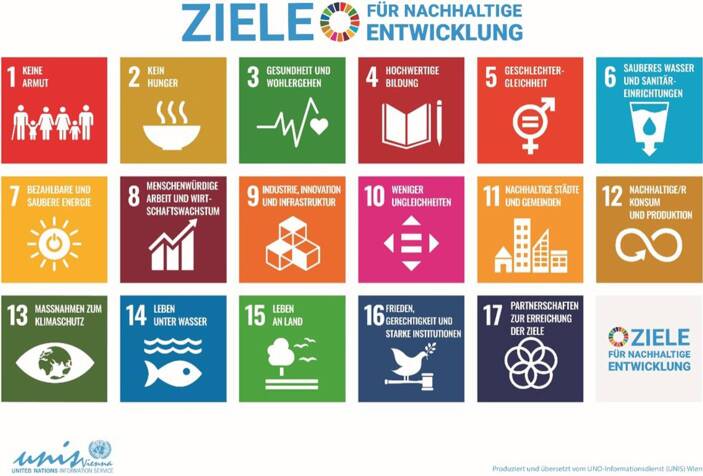

**Figure 2:**
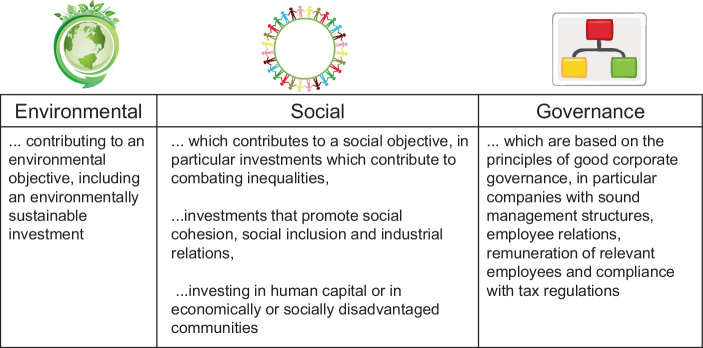

Eine Annäherung an den Begriff „Nachhaltigkeit“ wird daher in diesem Beitrag nicht weiter definitorisch vorgenommen, sondern erfolgt indirekt über Referenz-Leitbilder:

1. Nachhaltigkeit soll auf einer Maßnahmenebene im Sinne des Erreichens der 17 Nachhaltigkeitsziele der Agenda 2030 der Vereinten Nationen verstanden werden (siehe Abb. 1). Welche Aktivitäten tragen dazu bei, eines oder mehrere der (globalen) Nachhaltigkeitsziele zu erreichen oder stehen dem entgegen?
2. Nachhaltigkeit soll auf einer politisch-rechtlichen Ebene als dem Nachkommen des Paris Klimaabkommens verstanden werden. Sind die politischen Entscheidungen z. B. im Einklang mit einem Pfad, das Zwei-Grad-Ziel zu erreichen? Das Zwei-Grad-Ziel besagt, dass die globale Erwärmung langfristig auf höchstens zwei Grad Celsius (2 °C) über der globalen Mitteltemperatur vor der Industrialisierung beschränkt werden soll, woraus sich maximale CO_2_-Emissionen ableiten lassen.
3. Nachhaltigkeit soll aus ökonomischer Sicht – aufbauend auf den beiden anderen Ebenen – über Nachhaltigkeitsrisiken für einzelne Unternehmen oder Märkte insgesamt konkretisiert werden, insbesondere über die sog. ESG-Kriterien (environmental, social, governance, siehe Abb. 2).^9^

Da Risikomanagement in Versicherungsunternehmen und -gruppen einer (sektoralen) Regulierung und einer entsprechenden Beaufsichtigung durch Versicherungsaufsichtsbehörden unterliegt, ist diese Sichtweise auch diejenige auf regulatorischer Ebene. Die Identifikation und Bewertung der Nachhaltigkeitsrisiken kann als Nachhaltigkeits-Controlling verstanden werden, auf dem dann das eigentliche Nachhaltigkeits(risiko)management erfolgt.^10^ Das Monitoring dieser Managemententscheidungen, d. h. die Überwachung der Erreichung von Nachhaltigkeitszielen, schließt sich an und vervollständigt den Risikomanagement- bzw. Risiko-Controlling-Prozess in Bezug auf Nachhaltigkeit.

Die BaFin definiert Nachhaltigkeitsrisiken wie folgt:^11^

Nachhaltigkeitsrisiken […] sind Ereignisse oder Bedingungen aus den Bereichen Umwelt, Soziales oder Unternehmensführung […], deren Eintreten tatsächlich oder potenziell negative Auswirkungen auf die Vermögens‑, Finanz- und Ertragslage sowie auf die Reputation eines beaufsichtigten Unternehmens haben können.

Im betriebswirtschaftlichen Kontext wurde bisher häufig ein „Nachhaltigkeitsdreieck“ zur Veranschaulichung herangezogen, bei dem die Eckpunkte ökonomisch, ökologisch und sozial sind. Nachhaltigkeit will die Ziele Ressourcenschonung, z. B. Umweltschutz, wirtschaftliche Stabilität, Sozialverträglichkeit, soziale Verantwortung, soziale Gerechtigkeit und Gesundheitsverträglichkeit gleichzeitig und gleichwertig erreichen.^12^

Die Erreichung dieser Ziele in Kombination kann kompliziert werden, da der Zweck und die Grenzen der Verwirklichung sowohl die heutige als auch die zukünftige Generation nicht beeinträchtigen dürfen. Um Nachhaltigkeit für die gesamte Gesellschaft zu erreichen, ist es notwendig, Standards zu entwickeln. Diese Standards sollten auf jeden Bereich anwendbar sein.^13^

Bei den ESG-Kriterien wird gerade nicht die ökonomische Dimension gegen andere nicht-ökomische gestellt. Ein Ausgleich oder eine Kompensation ist konzeptionell nicht angelegt. Letztlich äußert sich jedoch das Eintreten oder Nicht-Eintreten von Nachhaltigkeitsrisiken in finanziellen Größen. Die Standardisierung hat daher bereits mit der Risikoidentifikation und Risikomessung im Sinne einer Bewertung zu beginnen, um die Ziele und die Zielerreichung transparent und über Referenzpunkte vergleichbar zu machen.

### Messbarkeit von Nachhaltigkeit?

Ein Blick in die Medien zeigt, dass Nachhaltigkeit ein wichtiger Aspekt für Unternehmen, Kunden und Märkte ist. Bereits im Jahr 2018 haben sich einige Versicherungsunternehmen aus wirtschaftlichen Gründen aus Kohleinvestitionen zurückgezogen. Wenn die Erderwärmung nicht auf weniger als zwei Grad begrenzt werden kann, muss mit teuren und sozial weitreichenden Folgen gerechnet werden, beim Überschreiten von sog. Kipppunkt evtl. sogar von unbeherrschbaren und auch nicht mehr versicherbaren Folgen. Unter anderem sind fossile Brennstoffe wie Kohle für den größten Teil der klimaschädlichen CO_2_-Emissionen verantwortlich.

Eines dieser Unternehmen, das Kohleinvestitionen ablehnt, ist die Munich Re. Die Munich Re investiert bereits heute nicht mehr in Aktien oder Anleihen von Unternehmen, die mehr als 30 % ihres Umsatzes mit Kohle erzielen oder Öl aus Ölsanden gewinnen.^14^ Außerdem versichert sie keine neuen Kohlekraftwerke/-minen und keine Anlagen zur Ölsandgewinnung mehr.^15^ Auch die Allianz hat schon 2018 ähnliche Nachhaltigkeitszielsetzungen in Hinblick auf Kohleinvestitionen kommuniziert.^9999^ Mittlerweile haben immer mehr (deutsche) Versicherungsunternehmen und -gruppen ihre Nachhaltigkeitsstrategien kommuniziert. Die Nachhaltigkeitspfade, auf denen sich die Unternehmen befinden, sind allerdings kaum vergleichbar (gewesen).^16^

Eine stärkere Vergleichbarkeit hat sich zuletzt dadurch ergeben, dass die Unternehmen sich gleichen Prinzipien unterwerfen (z. B. die UN Principles for Responsible Investment (UN PRI))^17^ oder sich zu Initiativen/Vereinigungen zusammenschließen, die einheitliche Ziele verfolgen. Zu nennen ist für letztere Entwicklung unter vielen die Net-Zero Asset Owner Alliance (NZAOA).^18^ Es bleibt aber weiterhin kritisch, dass Stakeholder, insbesondere auch Aufseher den Grad der Nachhaltigkeit und damit das Ausmaß von Nachhaltigkeitsrisiken verlässlich abschätzen können müssen. Dazu bedarf es regulatorischer Vorgaben, deren Entwicklungen im nachfolgenden Kap. 3 dargestellt werden.

## Regulatorische Entwicklungen

Im Folgenden werden regulatorische Entwicklungen zu Nachhaltigkeitsrisiken behandelt. Ausgewählte regulatorische Entwicklungen im zeitlichen Überblick ab 2015 bis heute zeigt Tab. 1. Für die Diskussion besonders relevant erachteter Ereignisse wird eine Differenzierung nach politischen Ebenen vorgenommen. Begonnen wird mit einer globalen Ebene, weil dort die weiteren Entwicklungen ihren Ausgangspunkt haben. Schwerpunkt wird auf die EU-Ebene gelegt, weil sie die Ebene darstellt, auf der die Versicherungsregulierung maßgeblich auch für deutsche Versicherungsunternehmen und -gruppen festgelegt wird. Die Ergänzung um die nationale Ebene fokussiert Deutschland.^19^

**Table 1:** 

Wann?	Wer?	Was?	Anmerkung
25.09.2015	Vereinte Nationen (2015b)	Transformation unserer Welt: die Agenda 2030 für nachhaltige Entwicklung	17 UN-Nachhaltigkeitsziele (vgl. Abb. 1)
15.12.2015	Vereinte Nationen (2015a)	Übereinkommen von Paris – Rahmenübereinkommen der Vereinten Nationen über Klimaänderungen („Pariser Klimaabkommen“)	22.04.2016 (EU unterzeichnet) 05.10.2016 (EU ratifiziert)
Dezember 2016	Europäische Kommission	Einsetzung einer hochrangigen Expertengruppe (HLEG) für nachhaltige Finanzen („sustainable finance“) mit Experten u. a. aus der Zivilgesellschaft, dem Finanzsektor und der Wissenschaft	Taxonomie
Juni 2017	TCFD (2017)	Recommendations of the Task Force on Climate-related Financial Disclosures (Empfehlungen der Task Force zu klimabezogenen Finanzinformationen)	Task Force ab 2016
31.01.2018	HLEG	Abschlussbericht der Expertengruppe für nachhaltige Finanzen („sustainable finance“)	–
März 2018	Europäische Kommission	Aktionsplan „Finanzierung nachhaltigen Wachstums“	–
Mai 2018	Europäische Kommission	Vorschlag eines Gesetzespaketes inkl. Taxonomie	Taxonomie
01.08.2018	Europäische Kommission (2018)	Formal request to EIOPA and ESMA for technical advices on potential amendments to, or introduction of, delegated acts with regard to the integration of sustainability risks and sustainability factors (Formales Ersuchen an EIOPA und ESMA für technische Empfehlungen zu möglichen Änderungen oder der Einführung delegierter Rechtsakte im Hinblick auf die Einbeziehung von Nachhaltigkeitsrisiken und Nachhaltigkeitsfaktoren)	Säule 1/2
Juli 2018	IAIS (2018)	Issues Paper on Climate Change Risks to the Insurance Sector (Themenpapier zu den Risiken des Klimawandels für den Versicherungssektor)	–
11/2018–01/2019	EIOPA (2018)	Consultation Paper on Technical Advice on the integration of sustainability risks and factors in the delegated acts under Solvency II and IDD (Konsultation zur Integration von Nachhaltigkeitsrisiken und -faktoren in die delegierten Rechtsakte unter Solvency II und IDD)	–
30.04.2019	EIOPA (2019b)	EIOPA’s Technical Advice on the integration of sustainability risks and factors in the delegated acts under Solvency II and IDD (Technische Empfehlungen EIOPAs zur Integration von Nachhaltigkeitsrisiken und -faktoren in die delegierten Rechtsakte unter Solvency II und IDD)	Säule 2 (Umsetzung durch Europäische Kommission 2021a)
30.09.2019	EIOPA (2019a)	Opinion on Sustainability within Solvency II (Stellungnahme zu Nachhaltigkeit in Solvency II)	Säule 1
04.12.2019	EIOPA (2019c)	Methodological Principles of Insurance Stress Testing (Methodische Grundsätze der Stress-Tests für Versicherungen)	Stress-Tests („first paper“)
11.12.2019	Europäische Kommission (2019b, c)	Vorstellung des europäischen Grünen Deal	–
20.12.2019	BaFin (2019)	Merkblatt zum Umgang mit Nachhaltigkeitsrisiken	Sektorübergreifend
03.03.2020	EIOPA (2020)	Methodological principles of insurance stress testing (Methodische Grundsätze der Stress-Tests für Versicherungen)	Stress-Tests
Juni 2020	–	Taxonomie-Verordnung (2020)	–
22.01.2021	GDV (2021)	Nachhaltigkeitspositionierung der deutschen Versicherungswirtschaft	–
Ab 10.03.2021	–	Offenlegungs-Verordnung (2019)	–
19.04.2021	EIOPA (2021a)	Opinion on the supervision of the use of climate change risk scenarios in ORSA (Stellungnahme zur Beaufsichtigung der Verwendung von Risikoszenarien für den Klimawandel im ORSA-Prozess)	Säule 2, speziell ORSA
21.04.2021	Europäische Kommission (2021a)	Änderung der Solvency II-Delegierten Verordnung (SII-DVO 2015) zur Einbeziehung von Nachhaltigkeitsrisiken in die Governance von Versicherungsunternehmen	Level 2 Säule 2 (geltend ab 02.08.2022)
Mai 2021	IAIS (2021)	Application Paper on the Supervision of Climate-related Risks in the Insurance Sector (Anwendungspapier zur Beaufsichtigung von klimabedingten Risiken im Versicherungssektor)	–
06.07.2021	Europäische Kommission (2021c)	Strategy for Financing the Transition to a Sustainable Economy (Strategie zur Finanzierung des Übergangs zu einer nachhaltigen Wirtschaft)	–
08.07.2021	EIOPA (2021d)	Methodological paper on potential inclusion of climate change in the Nat Cat standard formula (Methodisches Papier zur möglichen Berücksichtigung des Klimawandels in der Nat-Cat-Standardformel)	Säule 1
22.09.2021	Europäische Kommission (2021b)	Solvency II-Review-Richtlinie	Level 1 Säule 2 (ORSA) & EIOPA-Prüfauftrag (Säule 1)
November 2021	ISSB/IFRS-Stiftung	International Sustainability Standards Board (ISSB) (unter dem Dach der IFRS-Stiftung) mit der Aufgabe, globale Standards und Offenlegungsanforderungen für die Nachhaltigkeitsberichterstattung zu entwickeln (IFRS Sustainability Disclosure Standards (SDS))	Sitz Frankfurt, Montreal, etc.
Dezember 2021	EIOPA (2021c)	Sustainable Finance Activities 2022–2024	–
12/2021–02/2022	EIOPA (2021b)	Pilot Exercise on Climate Change Adaptation in Non-Life Underwriting and Pricing (Pilotübung zur Anpassung an den Klimawandel in der Zeichnung und Preisgestaltung von Nicht-Leben-Versicherungen)	–
27.01.2022	EIOPA (2022)	Methodological Principles of Insurance Stress Testing – Climate Change Component (Methodische Grundsätze für Stress-Tests für Versicherungen – Komponente Klimawandel)	Stress-Tests für mikro- und makroprudenzielle Zwecke („third paper“)

^a^Zu einer Übersicht klimawandelbezogener Aktivitäten von internationalen Regulierungsbehörden siehe auch The Geneva Association (2021, S. 34–35)

Aufgrund der Vielzahl der Initiativen auf den verschiedensten Ebenen und auch in unterschiedlichen Konstellationen in den letzten Jahren, insbesondere auch zuletzt, ist eine Auswahlentscheidung notwendig gewesen. Die bereits gestarteten Bemühungen zur Harmonisierung über Länder und über Sektoren sowie über Anwendungsfelder hinweg werden hoffentlich in Zukunft zu einheitlichen Ansätzen oder zumindest konsistenten und kompatiblen Daten und/oder Vorgehensweisen führen.

Auch, wenn die Nachhaltigkeitsberichterstattung mittlerweile z. T. etabliert ist und ein starker Treiber für Nachhaltigkeitsentwicklungen auch im Finanzdienstleistungssektor ist, sollen die entsprechenden regulatorischen Entwicklungen im Folgenden außen vor gelassen werden.^20^

Die EU-Taxonomie ist dabei sicherlich ein entscheidender konzeptioneller Baustein, insbesondere würde sie erlauben, dass die Berichterstattung und die Daten in gewissem Maße standardisiert (öffentlich) zur Verfügung stehen könnten, evtl. sogar über einen einzigen digitalen Zugangspunkt. Dass standardisierte umfangreiche Daten im Detail bereits in nächster Zukunft beispielsweise für Stress-Tests in benötigter Granularität zur Verfügung stehen werden, ist aber unwahrscheinlich. Die Zielsetzung, solche Daten zur Verfügung stellen, bleibt davon unberührt und mittelfristig auch relevant für die Weiterentwicklungen zur Quantifizierung von Nachhaltigkeitsrisiken.

### Regulatorische Entwicklungen auf globaler Ebene

#### Nachhaltigkeitsziele

2015 wurden von den Vereinten Nationen 17 Ziele für eine nachhaltige Entwicklung im Rahmen einer Agenda 2030 verabschiedet.^21^ Sie bilden einen Referenz-Rahmen für politische und wirtschaftliche Aktivitäten und erlauben eine Konkretisierung von Nachhaltigkeit, die auch z. B. in Unternehmen hilfreich sein kann, eine Nachhaltigkeitsstrategie zu entwickeln (vgl. Abb. 1). Wenn auch keine Regulierung im eigentlichen Sinne, haben diese Nachhaltigkeitsziele einen global bindenden Charakter.

#### Pariser Klimaabkommen

Teil der Entwicklung auf globaler Ebene ist das Pariser Abkommen, das ebenfalls 2015 geschlossen wurde.^22^ Zum ersten Mal wurden alle Nationen zusammengebracht, um Anstrengungen zur Bekämpfung des Klimawandels zu unternehmen. Ziel ist es, den globalen Temperaturanstieg zu verhindern und die Länder im Umgang mit den Auswirkungen des Klimawandels zu stärken. Die wichtigsten Aspekte des Abkommens sind u. a.:^23^

- Langfristiges Temperaturziel, d. h. Begrenzung des globalen Temperaturanstiegs auf unter 2 °C (sog. Zwei-Grad-Ziel), möglichst auf nur 1,5 °C
- Abschwächung, indem verbindliche Verpflichtungen festgelegt werden, die alle fünf Jahre vorgelegt werden müssen
- Senken und Speichern von Treibhausgasen
- Freiwillige Zusammenarbeit/Markt- und nicht-marktbasierte Ansätze
- Anpassung und internationale Zusammenarbeit
- Verlust und Schaden, Ansätze zur Bewältigung der negativen Auswirkungen des Klimawandels
- Finanzierung, Technologie und Aufbau von Kapazitäten
- Erziehung zum Klimawandel, Ausbildung, Sensibilisierung der Öffentlichkeit, Beteiligung der Öffentlichkeit und Zugang der Öffentlichkeit zu Informationen
- Transparenz, Umsetzung und Einhaltung.

Hervorzuheben ist, dass im Pariser Klimaabkommen auch der Ausgangspunkt für eine nachhaltige Finanzwirtschaft („sustainable finance“) gelegt ist. Weit weniger bekannt und zunächst auch weniger beachtet als z. B. das Zwei-Grad-Ziel ist die Festlegung in Art. 2 Abs. 1 c), dass „die Finanzmittelflüsse in Einklang gebracht werden mit einem Weg hin zu einer hinsichtlich der Treibhausgase emissionsarmen und gegenüber Klimaänderungen widerstandsfähigen Entwicklung.“^24^

Hier gibt das Übereinkommen das Ziel vor, entschlossen gegen Klimaänderungen vorzugehen, unter anderem indem nicht weiter in Kohle investiert werden soll. Die unscheinbar anmutende Formulierung hat sich mittlerweile zu einer starken Bewegung unter dem Begriff „sustainable finance“ entwickelt, die vor allem auch regulatorisch vorangetrieben wird und beispielsweise von der EU aufgegriffen wurde.

### Regulatorische Entwicklungen auf EU-Ebene

Im Folgenden werden ausgewählte regulatorische Entwicklungen auf EU-Ebene mit Bezug zu Nachhaltigkeitsrisiken näher betrachtet (in zeitlicher Abfolge).

#### EIOPA-Stellungnahme zu Nachhaltigkeit in Solvency II

Im August 2018 wurde EIOPA (wie auch ESMA) von der Europäischen Kommission um eine Stellungnahme zu Nachhaltigkeit in Solvency II gebeten,^25^ wobei der Schwerpunkt auf Aspekten des Klimaschutzes lag. EIOPA hat dazu die folgenden Referenz-Festlegungen betrachtet:^26^

Bei der Behandlung des Themas „Klimarisiken und klimawandelbedingte Risiken“ hat EIOPA berücksichtigt, dass „Klimarisiken“ alle Risiken umfassen sollen, die sich aus durch den Klimawandel verursachten Trends oder Ereignissen ergeben, d. h. klimawandelbedingte Risiken. Dazu gehören extreme Wetterereignisse wie Naturkatastrophen, aber auch allgemeinere Klimatrends wie der allgemeine Temperaturanstieg, der Anstieg des Meeresspiegels oder klimabedingte Zwangsmigrationen, die sich auf die (Rück‑)Versicherungstätigkeit auswirken könnten.

Hinsichtlich der Bewertung von Vermögenswerten war EIOPA der Ansicht, dass weitere Verbesserungen bei der Verfügbarkeit und Qualität der für ihre Bewertung relevanten Informationen erforderlich sind, um sicherzustellen, dass die Marktpreise die Nachhaltigkeitsrisiken und -faktoren besser widerspiegeln können. Darüber hinaus sollten Szenarioanalysen angewandt werden, um Unsicherheiten im Zusammenhang mit dem Klimawandel zu bewerten und sicherzustellen, dass alle ESG-Kriterien übergreifend sind.

Die EIOPA schlägt außerdem vor, Szenarioanalysen zu nutzen, um die Angemessenheit des besten Schätzwerts („best estimate“) unter Berücksichtigung der mit dem Klimawandel verbundenen Risiken sicherzustellen, der Teil der marktkonsistenten Bewertung der versicherungstechnischen Verbindlichkeiten ist. Die Unternehmen sollten sowohl historische Daten als auch die wissenschaftliche Literatur verwenden. Insgesamt sollten die Unternehmen sicherstellen, dass ihre historischen Verlustdaten auf dem neuesten Stand sind, mögliche Ereignisse berücksichtigen, die von den historischen Verlustdaten des Unternehmens nicht erfasst werden, und eine zukunftsorientierte Katastrophenmodellierung entwickeln und anwenden sowie Stress-Tests oder Szenarioanalysen durchführen.^27^

Mit Blick auf die Underwriting-Praktiken war EIOPA der Meinung, dass Versicherungsunternehmen zur Anpassung an den Klimawandel und zu dessen Abschwächung beitragen sollten. Ein einschlägiges Beispiel ist das „Impact Underwriting“, welches die Entwicklung neuer Versicherungsprodukte, Anpassungen in der Gestaltung und Preisgestaltung der Produkte und die Zusammenarbeit mit den Behörden umfasst, ohne die versicherungsmathematischen, risikobasierten Grundsätze der Risikoselektion und Preisgestaltung zu missachten.

Im Hinblick auf die Kapitalanforderungen musste lt. EIOPA jede Änderung auf einem nachgewiesenen Risiko beruhen, das sich vom Status quo unterscheidet. Das zugrundeliegende Risiko ist hier der Ausgangspunkt. Eines davon ist das Marktrisiko. In diesem Zusammenhang ist es z. B. notwendig, das Risikoprofil verschiedener Arten von Immobilien zu berechnen und über Daten zu verfügen, die mehr als einen Konjunkturzyklus abdecken. Ein weiteres Risiko ist das Naturkatastrophenrisiko. Hier schlägt EIOPA vor, dass die Modellierer von Katastrophenrisiken ihre Analysen über die möglichen Auswirkungen des Klimawandels ausweiten und die Ergebnisse dieser Analysen in ihre Modelle einfließen lassen sollten.

Was die internen Modelle betrifft, so wird vorgeschlagen, nicht nur historische Daten zu verwenden, sondern einen stärker zukunftsorientierten Ansatz zu verfolgen und spezifische und konsistente Szenarien anzuwenden.

Die letzten Themen sind Herausforderungen bei der Integration von Überlegungen zu nachhaltigen Finanzen in die Anforderungen der Säule 1 und Vorschläge für das weitere Vorgehen. EIOPA sah keine Notwendigkeit, den Zeithorizont zu ändern, schlug aber vor, Instrumente wie Szenarioanalysen und Stress-Tests in Säule 2 zu verwenden, um die Auswirkungen des Klimawandels zu erfassen. EIOPA ist der Meinung, dass die Szenarien auf das Risikoprofil der Unternehmen zugeschnitten sein sollten. Bei der Entwicklung dieser Szenarien sollten die Unternehmen die folgenden Fragen berücksichtigen:^28^

- Welche Aktivitäten (Kapitalanalage, Underwriting, strategische Planung, Entwicklung neuer Produkte, usw.) könnten von klimawandelbedingten Risiken betroffen sein?
- Wie wesentlich sind diese Risiken für die betroffenen Aktivitäten?
- Welche Zeithorizonte sollten berücksichtigt werden?
- Welche Szenarien sollten in Betracht gezogen werden?
- Welche Daten und Instrumente sind für die Durchführung der Szenarioanalyse verfügbar?

#### Einbeziehung von Nachhaltigkeitsrisiken in die Solvency II-Delegierte Verordnung

Mit einer delegierten Verordnung zur Änderung der Solvency II-Delegierten Verordnung (SII-DVO 2015) führte die Europäische Kommission eine Definition von Nachhaltigkeitsrisiken auf Level 2 von Solvency II ein.^29^ Als „Nachhaltigkeitsrisiko“ wird definiert „ein Ereignis oder eine Bedingung in den Bereichen Umwelt, Soziales oder Unternehmensführung, dessen beziehungsweise deren Eintreten tatsächlich oder potenziell negative Auswirkungen auf den Wert der Investition oder auf den Wert der Verbindlichkeit haben könnte“ (Art. 1 Nr. 55c). Es erfolgt hier also ebenfalls eine Definition von Nachhaltigkeit über die ESG-Kriterien (s. 2.1) sowohl auf der Aktiv- als auch Passivseite der Solvency II-Bilanz.

Die so definierten Nachhaltigkeitsrisiken sind nun explizit beim Grundsatz der unternehmerischen Vorsicht („prudent person principle“) bei der Kapitalanlage zu berücksichtigen (Art. 275a SII-DVO (2015)). Dies stellt in gewisser Weise nur eine Klarstellung dar, da bisher auch schon alle relevanten Risiken zu berücksichtigen waren. Aus der nun nicht mehr nur impliziten Berücksichtigung folgen jedoch zumindest höhere Anforderungen an den Nachweis der Einhaltung der Kapitalanlagevorschriften, beispielsweise durch entsprechend erweiterte Checklisten und Dokumentation.

Außerdem sind die Nachhaltigkeitsrisiken nun im Risikomanagementsystem und beim Gesamtsolvabilitätsbedarf ebenfalls explizit zu berücksichtigen (Art. 260 bzw. 262 SII-DVO (2015)). Die Ermittlung und Bewertung von Nachhaltigkeitsrisiken wird analog sich abzeichnender Risiken (sog. emerging risks) explizite Aufgabe der Risikomanagement-Funktion (Art. 269 SII-DVO (2015)). Auch wenn die Säule 2-Vorschriften qualitatives Risikomanagement im Kern beschreiben, klingt hier auch die Notwendigkeit der Quantifizierung von Nachhaltigkeitsrisiken an.

Um sicherzustellen, dass Nachhaltigkeitsrisiken angemessen gemanagt werden, müssen zusätzlich die Vergütungs-Leitlinien nun Angaben dazu enthalten, wie der Einbeziehung von Nachhaltigkeitsrisiken in das Risikomanagementsystem Rechnung getragen wird (Art. 275 SII-DVO (2015)).

Die Europäische Kommission folgte damit den EIOPA-Vorschlägen.^30^ Diese neuen Level 2-Vorschriften werden ab August 2022 für Versicherungsunternehmen und -gruppen unter Solvency II-Aufsicht gelten. Wegen der Rechtssetzung als delegierte Verordnung gelten sie in den EU-Mitgliedstaaten unmittelbar und bedürfen keiner Umsetzung in nationales Recht.

#### EIOPA zur Überarbeitung des Naturkatastrophen-Moduls in der Standardformel

In einer Veröffentlichung erörtert EIOPA die bisher für die Kalibrierung des SCR für Naturkatastrophen verwendete Methodik im Standardansatz und stellt Gefahren und Länder vor, die vom Klimawandel wesentlich betroffen sein könnten.^31^ Zu konstatieren ist, dass das höhere Bewusstsein für Nachhaltigkeitsrisiken sowie anhaltend hohe Schäden durch Naturkatastrophen (wie 2021 im Ahrtal) eine Neubewertung der bestehenden Parameter erforderten.

Häufigkeit und Schwere von Naturkatastrophen werden aufgrund des Klimawandels zunehmen. Um den Schutz der Versicherungsnehmer und die Stabilität des Versicherungsmarktes zu gewährleisten, sollten das SCR für das versicherungstechnische Risiko von Naturkatastrophen die erwarteten Auswirkungen des Klimawandels widerspiegeln. Es werden technische Vorschläge erläutert, wie der Klimawandel in die NatCat-SCR-Kalibrierung in der Standardformel zukünftig besser und zeitgerecht einbezogen werden kann.

Eine Umsetzung dieser Vorschläge könnte auch im Kontext des Solvency II-Review vorgenommen werden, der im Folgenden dargestellt wird.

#### Vorschläge der Europäischen Kommission zu Nachhaltigkeit im Solvency II-Review

Eine der Zielsetzungen der Europäischen Kommission bei der Überprüfung der Solvency II-Richtlinie (SII-RL 2009) ist neben der Finanzierung der wirtschaftlichen Erholung nach der Pandemie Mittel durch geringere Kapitalanforderungen und verbesserte langfristige Investitionsmöglichkeiten in Richtung des europäischen Grünen Deals zu kanalisieren.

Die Vorschläge der Europäischen Kommission zum Solvency II-Review^32^ beinhalten auch Vorschläge hinsichtlich Nachhaltigkeitsrisiken. Diese sind nun explizit in die unternehmenseigene Risiko- und Solvabilitätsbeurteilung (ORSA) einzubeziehen (Art. 45a SIIR-RL). Versicherungsunternehmen sollen über anzustellende Klima-Szenarien alle wesentlichen klimawandelbezogenen Risiken ermitteln und die Auswirkungen langfristiger Klimawandel-Szenarios auf ihre Geschäftstätigkeit bewerten.^33^ Dies impliziert auch eine Ausweitung des zeitlichen Horizonts der ORSA-Berichterstattung über die bisherigen 3 bis 5 Jahre („Planungshorizont“) hinaus.

EIOPA erhält zudem den Auftrag, bis 2023 eine spezielle aufsichtsrechtliche Behandlung von Risiken im Zusammenhang mit Vermögenswerten oder Tätigkeiten zu prüfen, die im Wesentlichen mit ökologischen und/oder sozialen Zielen verbunden sind (Art. 304a SIIR-RL). Damit wird die Diskussion zu sog. „green supporting“- bzw. „brown penalising“-Faktoren aufgegriffen. Zudem soll EIOPA mindestens alle drei Jahre den Umfang und die Kalibrierung der Parameter der Standardformel für das Naturkatastrophenrisiko überprüfen (vgl. 3.2.3). Nachhaltigkeitsrisiken führen von daher nicht zu einem eigenen SCR-Risikomodul, sondern sind in den bestehenden Risikomodulen abzubilden (vgl. 4.3.1).

EIOPA hat die Vorschläge der Europäischen Kommission zu Nachhaltigkeit im Solvency II-Review begrüßt.^34^

#### EIOPA-Pilotübung Nicht-Leben

Ende 2021 startete EIOPA eine freiwillige Pilotübung („pilot exercise“), um besser zu verstehen, wie Versicherungsunternehmen klimabezogene Anpassungsmaßnahmen in Nicht-Leben-Versicherungsprodukte integrieren und um die Angemessenheit der entsprechenden aufsichtsrechtlichen Behandlung dieser Versicherungsprodukte zu bewerten. Die Zeichnungs- und Preispolitik der Versicherungsunternehmen soll dazu näher betrachtet werden. Die Möglichkeit beizutragen, stand allen Interessierten bis Februar 2022 offen; mit den Stakeholdern sollen ab März 2022 Interviews geführt werden.

In den nächsten drei Jahren bis 2024 wird „Sustainable Finance“ weiterhin eine hohe strategische Priorität für die Arbeit von EIOPA haben.^35^

### Entwicklungen auf nationaler Ebene

Im Folgenden werden ausgewählte regulatorische Entwicklungen auf nationaler Ebene vorgestellt, wobei der Fokus auf Deutschland liegt. Auch in anderen EU- und Nicht-EU-Ländern sind Aufsichtsbehörden in Hinblick auf Nachhaltigkeit aktiv geworden.^36^ Angesichts der Klimakrise als globaler Herausforderung ist die Fragmentierung der Aktivitäten vielleicht verwunderlich, es dürfte aber mit zunehmender Dauer zu einer Koordinierung unter den Aufsichtsbehörden kommen, die zu mehr Kohärenz der Vorgaben auch in diesem Feld führen sollte. Insbesondere für internationale Versicherungsgruppen und Finanzkonglomerate ist es von hoher Bedeutung, dass Investitionen in z. B. Datenerfassung und Berichtsformate nicht mehrfach getätigt werden müssen.

Für Gruppen unter Solvency II-Gruppenaufsicht haben die EU-Vorschriften eine Ausstrahlungswirkung, da Säule 2-Vorgaben (ORSA-Prozess!) auch auf Gruppen-Ebene mutatis mutandis gelten.

#### BaFin-Merkblatt zum Umgang mit Nachhaltigkeitsrisiken

Die Bundesanstalt für Finanzdienstleistungsaufsicht (BaFin) hat Ende 2019 ein sog. Merkblatt zum Umgang mit Nachhaltigkeitsrisiken veröffentlicht,^37^ das zuvor zwischen September und November 2019 öffentlich konsultiert worden war. Es ist sektorübergreifend konzipiert und beschreibt Anforderungen an beaufsichtigte Unternehmen, wie Kreditinstitute, Versicherungsunternehmen und Kapitalverwaltungsgesellschaften.

Es behandelt Nachhaltigkeitsrisiken im Hinblick auf den aufsichtlichen Prüfungsprozess und enthält Good-Practice-Ansätze sowie Beispiele. Zudem werden Ergänzungen zu den Mindestanforderungen an das Risikomanagement formuliert.

Die BaFin stellt u. a. in den folgenden Bereichen Anforderungen an den Umgang mit Nachhaltigkeitsrisiken:^38^

- Strategien der beaufsichtigten Unternehmen,
- Verantwortungsvolle Unternehmensführung („Tone at the top“),
- Geschäftsorganisation („Governance“),
- Risikomanagement,
- Stress-Tests einschließlich Szenarioanalysen,
- Auslagerung/Ausgliederung (Outsourcing),
- Gruppensachverhalte und
- Verwendung von Ratings.

Die Vorgaben sind also überwiegend an das qualitative Risikomanagement gerichtet, bei Versicherungsunternehmen und -gruppen unter Solvency II-Aufsicht demnach in Säule 2.

#### BaFin-Umfrage zu Sustainable Finance (nachhaltige Finanzwirtschaft)

Bereits 2018 hatte die BaFin eine Umfrage zu Kapitalanlagen und Nachhaltigkeit durchgeführt und Ergebnisse dazu veröffentlicht.^39^ 2021 erfolgte eine erneute BaFin-Umfrage zum Thema Sustainable Finance (nachhaltige Finanzwirtschaft), deren aggregierten Ergebnisse als Sachstandsbericht veröffentlicht wurden.^40^ Die Umfrage zielt auch auf die „Umsetzung“ des BaFin-Merkblatts zum Umgang mit Nachhaltigkeitsrisiken durch Versicherer und Pensionsfonds (vgl. 3.3.1). Die BaFin sieht den Versicherungssektor auf einem guten Weg, wobei der Weg noch lang sei.^41^

Während die Bedeutung von Nachhaltigkeit in den teilnehmenden Unternehmen erkannt und auch entsprechende Verantwortlichkeiten festgelegt sind, ist beispielsweise die Bewertung von Nachhaltigkeitsrisiken lt. BaFin noch nicht sehr fortgeschritten: „Die Bewertung von Nachhaltigkeitsrisiken beruht in mehr als drei Vierteln der Unternehmen bislang auf Expertenschätzungen. Quantitative Methoden sind noch die Ausnahme, da in den meisten Unternehmen entsprechende Datengrundlagen noch nicht vorhanden sind.“^42^

Außerdem „setzt bislang nicht einmal ein Viertel der Unternehmen nachhaltigkeitsbezogene Stresstests und Szenarioanalysen ein. Weniger als die Hälfte der Umfrageteilnehmer gibt an, entsprechende Stresstests zumindest vorzubereiten.“^43^

Die BaFin thematisiert diese Diskrepanz und sieht „dringenden Nachholbedarf“ bei der Verwendung interner Stress-Tests und Szenarioanalysen, insbesondere, weil die BaFin klimawandelbezogene Szenarioanalysen im ORSA von Versicherungsunternehmen, für die Solvency II gilt, schon ab diesem Jahr [= 2022] erwartet, sofern diese Risiken für die Unternehmen wesentlich sind.^44^ Sie greift damit eine EIOPA-Empfehlung auf, bei der Durchführung ihrer unternehmenseigenen Risiko- und Solvabilitätsbeurteilung (ORSA) auch Szenarien zu Klimawandelrisiken zu betrachten, wenn diese Risiken wesentlich für sie sind.^45^ Zu erinnern ist daran, dass die Maßnahmen lediglich proportional zum Risikoprofil sein müssen.

## Szenarioanalysen zu Nachhaltigkeitsrisiken

### Szenarioanalysen als Risikomanagement-Instrument

Die Szenarioanalyse stellt eine mögliche zukünftige Situation dar, einschließlich der Entwicklungspfade, die zu einer zukünftigen Entwicklung führen. Darüber hinaus beschreibt ein Szenario die Entwicklungen, die Dynamik und die treibenden Kräfte, aus denen sich eine bestimmte Vision der Zukunft in konsistenter Weise ergibt („Zukunftsbild“). In Verbindung mit der Durchführung einer großen Anzahl von Szenarien bietet diese Methodik ein Spektrum möglicher Entwicklungen in einem zu analysierenden Gebiet.

Im Folgenden werden beispielhaft Szenarien, die sich auf Nachhaltigkeitsrisiken beziehen, vorgestellt.

1. **Knappheit der Ressourcen**
  Wegen Problemen in der Lieferkette kommt es zu einer Verknappung der verwendeten Ressourcen. Infolgedessen muss sich das Unternehmen nach einem Ersatz umsehen. Dieser abrupte Wechsel beeinflusst auch die Qualität der Produkte, was zur Unzufriedenheit der Kunden führt.
2. **Wettbewerb**
  In diesem Szenario arbeitet die Konkurrenz im Vergleich zu dem betrachteten Unternehmen nachhaltig. Infolgedessen verliert das Unternehmen Kundinnen und Kunden, da diese eher bereit sind, bei der Konkurrenz zu kaufen. Die Kundenaufträge gehen zurück und damit auch die Gewinne des Unternehmens.
3. **Reputation („Greenwashing“)**
  Ein Unternehmen behauptet, nur mit umweltfreundlichen Ressourcen zu produzieren. Nach einer Untersuchung finden Fachleute heraus, dass einige der verwendeten Ressourcen umweltschädlich sind. Der Name des Unternehmens taucht in allen Zeitungen auf, was dem Ruf schadet.

Diese Szenarien zeigen exemplarisch die Komplexität, die Nachhaltigkeitsrisiken innewohnen kann und dass aus Nachhaltigkeit selbst wiederum Risiken resultieren können (Beispiel 3 oben). Eine einfache Modellierung über einzelne Parameter, wie sie beispielsweise bei Sensitivitäten bei finanziellen Risiken, erfolgt, kann dem letztlich nicht genügen.

### Berücksichtigung von Nachhaltigkeitsrisiken im ORSA-Prozess

Versicherungsspezifisch werden nun pragmatische, konzeptionelle Herangehensweisen vorgestellt, die erste Schritte zu weitergehenden Modellierungen sein könnten, wobei diese im Risikomanagement-Prozess gesehen werden, der aufsichtsrechtlich die unternehmensindividuelle Risiko- und Solvenzbeurteilung (ORSA) darstellt. Bei Szenarioanalysen im ORSA-Prozess kann die Differenzierung von Nachhaltigkeitsrisiken in physische Risiken und Transitionsrisiken genutzt werden.

Die BaFin definiert physische Risiken und nennt Beispiele für physische Risiken, wobei sie wiederum kurzfristige/akute Ereignisse auf der einen Seite und in langfristige/chronische Veränderungen auf der anderen Seite unterscheidet: „Physische Risiken ergeben sich sowohl im Hinblick auf einzelne Extremwetterereignisse und deren Folgen (Beispiele: Hitze- und Trockenperioden, Überflutungen, Stürme, Hagel, Waldbrände, Lawinen) als auch in Bezug auf langfristige Veränderungen klimatischer und ökologischer Bedingungen (Beispiele: Niederschlagshäufigkeit und -mengen, Wetterunbeständigkeit, Meeresspiegelanstieg, Veränderung von Meeres- und Luftströmungen, Übersäuerung der Ozeane, Anstieg der Durchschnittstemperaturen mit regionalen Extremen).“^46^

Für beide Kategorien innerhalb der physischen Risiken sind relevante Szenarien für das Unternehmen herauszufiltern (z. B. für die eigenen Immobilien). Für die jeweiligen Risiken sind dann entsprechende Zeithorizonte für die jeweiligen Szenarien zu wählen.

Transitionsrisiken (auch: transitorische Risiken, Übergangsrisiken) sind demgegenüber definiert als Risiken „im Zusammenhang mit der Umstellung auf eine klimaneutrale Wirtschaft: Politische Maßnahmen können zu einer Verteuerung und/oder Verknappung fossiler Energieträger führen (Beispiele: Kohleausstieg, CO2-Steuer, Emissionszertifikate) oder zu hohen Investitionskosten aufgrund erforderlicher Sanierungen von Gebäuden und Anlagen. Neue Technologien können bekannte verdrängen (Beispiel: Elektromobilität), veränderte Präferenzen der Vertragspartner und gesellschaftliche Erwartungen können nicht angepasste Unternehmen gefährden.“^47^

Für die genannten Beispiele ist es schon deutlich schwieriger, entsprechende Szenarien zu entwickeln, da sie von politischen Entscheidungen abhängen können, die z. B. früher oder später (evtl. erst unter einer neuen Regierung) getroffen werden. Hinzu kommt, dass Interdependenzen zwischen physischen Risiken und Transitionsrisiken bestehen: Eine starke Zunahme der physischen Risiken würde eine abruptere Umstellung der Wirtschaft erfordern, was wiederum zu höheren Transitionsrisiken führt.^48^

Hinzu kommen Szenarien für soziale und Governance-Aspekte, die eine weitere Herausforderung neben der ökologischen Dimension darstellen. Hier sind Schäden auch vor allem als Reputationsrisiken denkbar.^49^

Ein einfacher Ansatz ist, auch Ausschlusskriterien^50^ oder Limite, z. B. für nachhaltige Investitionen, zu betrachten (siehe Tab. 3 im Anhang) und zu prüfen, ob solche Investitionen im eigenen Bestand vorhanden sind. Für diese können dann spezifische Szenariobetrachtungen durchgeführt werden, auch wenn ihr Anteil oder Wert eher gering ausfällt. Daraus ergeben sich wertvolle Einsichten in evtl. notwendige Anpassungen der Kapitalanlagestrategie.

Dies kann als eine Form des „Alignment“ verstanden werden. Die meint, die eigene Geschäftstätigkeit an politischen Zielsetzungen auszurichten, um Wachstumschancen zu nutzen bzw. die negativen Folgen der Durchsetzung dieser politischen Ziele möglichst zu vermeiden.^51^

In der Praxis häufig sinnvoll wäre beispielsweise eine Angleichung an die Ziele des Pariser Klimaabkommens, wozu Unternehmen sich selbst einen Dekarbonisierungspfad vorgeben. Dies bedeutet, dass die Produktion von Waren oder die Erbringung von Dienstleistungen bzw., im Falle des Finanzsektors, die Bereitstellung von Kapital, in bestimmten Zeitschritten mit kontinuierlich sinkenden Treibhausgasemissionen verbunden sein muss, so dass das Unternehmen am Ende (netto) nicht mehr zur Klimaerwärmung beiträgt.^52^ Referenz-Leitbild können auch sein die nachhaltigen Entwicklungsziele der Vereinten Nationen (vgl. 2.1) oder auch die Taxonomie-Verordnung (2020) als mögliche Optionen für strategische Festlegungen z. B. hinsichtlich einer Strategic Asset Allocation (SAA).^53^

Für unternehmensindividuelle Stress-Tests bzw. Szenarioanalysen für Nachhaltigkeitsrisiken können zum Beispiel die vom Network for Greening the Financial System (NGFS) weiterentwickelten Szenarien dienen,^54^ wobei die eingeschränkten Annahmen der Modelle zu berücksichtigen wären (z. B. wenn Extremwetterereignisse, Meeresspiegelanstieg und soziale Auswirkungen nicht eingerechnet sind).^55^

### Berücksichtigung von Nachhaltigkeitsrisiken im Standardansatz

Die Kapitalanforderungen unter Solvency II sind zum einen die Solvency Capital Requirement (SCR) und das Minimum Capital Requirement (MCR). Da die Berechnung des MCR nicht ausreichend risikobasiert erfolgt, erscheint keine weitere Berücksichtigung von Nachhaltigkeitsrisiken erforderlich. Dadurch, dass das MCR in einer vorgegebenen Spannbreite gemessen am SCR liegt, ergäbe sich aus der Berücksichtigung von Nachhaltigkeitsrisiken im SCR auch eine implizite Berücksichtigung im MCR. Insbesondere würde eine deutliche Erhöhung des SCR auch zu einer Erhöhung des MCR führen.

In Säule 2 ist eine Berechnung eines Gesamtsolvabilitätsbedarf verlangt, die auch dazu dient, die Annahmen der SCR-Berechnungen in Säule 1 zu validieren. Naheliegend ist daher auch für die Säule 2-Berechnungen das Risikomaß und die grundsätzliche Berechnungsstruktur beizubehalten und nur bei unternehmensspezifischen Besonderheiten davon abzuweichen und andere Ansätze zu wählen. Mit der Änderung der Solvency II-Delegierten Verordnung (SII-DVO 2015) ist die explizite Berücksichtigung von Nachhaltigkeitsrisiken bei der Ermittlung des Gesamtsolvabilitätsbedarfs auch regulatorisch vorgegeben.

Als Risikomaß für die SCR-Berechnung dient unter Solvency II der Value at Risk (VaR) zum Konfidenzniveau von 99,5 % auf Ein-Jahres-Sicht (sog. SCR-Kalibrierung): SCR := |VaR_99.5_(X)|. Diese Risiko-Festlegung ist auch für die einzelnen SCR der einzelnen Risikokategorien (Risikomodule bzw. Risikosubmodule) vorgesehen. Innerhalb der Risikomodule erfolgt die Risikoaggregation durch Korrelationsfaktoren, die die lineare (statistische) Abhängigkeit zwischen jeweils zwei SCR beschreibt.^56^ Für die fünf Risikomodule auf oberste Ebene ergibt sich folgende SCR-Aggregation:^57^

$$\text{BSCR}=\sqrt{\sum _{ij}\text{Corr}_{ij}\cdot \mathrm{SCR}_{i}\cdot \mathrm{SCR}_{j}}+\mathrm{SCR}_{\text{intangibles}}$$

Vereinfachend sollen an dieser SCR-Kalibrierung und -Aggregation („Standardformel“) die Alternativen der Berücksichtigung von Nachhaltigkeitsrisiken betrachtet werden.

#### Standardformel: separates Risikomodul

**Figure 3:**
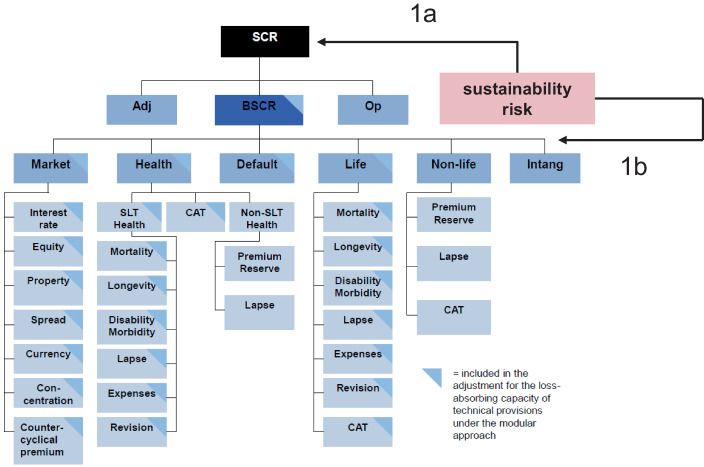

Eine erste Idee könnte sein, das SCR anzupassen, indem man das Nachhaltigkeitsrisiko als Ganzes hinzufügt, wobei zuvor das Nachhaltigkeits-SCR in einem separaten Risikomodul ermittelt wurde (vgl. Variante 1a in Abb. 3).

Solvency II berechnet den SCR jedoch mit einer Kalibrierung eines VaR mit einem Konfidenzniveau von 99,5 % über einen Zeithorizont von einem Jahr. Die Frage wäre insbesondere, ob bzgl. physischen und transitorischen Risiken der Klimawandel als 200-Jahres-Ereignis betrachtet werden kann?

Die Herausforderung ist zudem, dass, um eine Doppelberücksichtigung zu vermeiden, in den anderen SCR keine Nachhaltigkeitsrisiken enthalten sind. Insbesondere erscheint dies dann zu erfordern, bei Naturkatastrohen zu unterscheiden, ob diese Klimawandel-bedingt sind oder nicht. Auch, wenn die sog. Attributionsforschung genau dazu Ansätze entwickelt hat, erscheint dies wenig praktikabel auf granularer Ebene der Schäden.^58^ Vorteil wäre, dass keine Korrelationen zu anderen SCR betrachtet werden müssten und die bestehenden, sofern risikobasiert, beibehalten werden könnten.

Die zweite Idee könnte darin bestehen, das Nachhaltigkeitsrisiko ebenfalls als Ganzes, aber als zusätzliches Risikomodul auf Ebene der anderen Hauptrisikomodule hinzuzufügen (Variante 1b). Dies würde die Festlegung von Korrelationsfaktoren mit den anderen SCR erfordern. Möglich wäre es, in den anderen SCR enthaltene Nachhaltigkeitsrisiken dort zu belassen und nur die nicht bereits berücksichtigten in dem separaten Risikomodul mitaufzunehmen. Umgekehrt könnte eine gewisse Korrektur über die Korrelationsfaktoren erfolgen.

Beide Varianten (1a oder 1b, nicht in Kombination!) lösen aber nicht die Problematik der Bestimmung des Nachhaltigkeitsrisikos insgesamt aus ganz verschiedenen Risiken sowohl auf der Aktiv- als auch auf der Passivseite. Zudem ist dies nur mit sehr groben Annahmen über Abhängigkeitsstrukturen möglich.

#### Standardformel: Integration in Risikomodule

**Figure 4:**
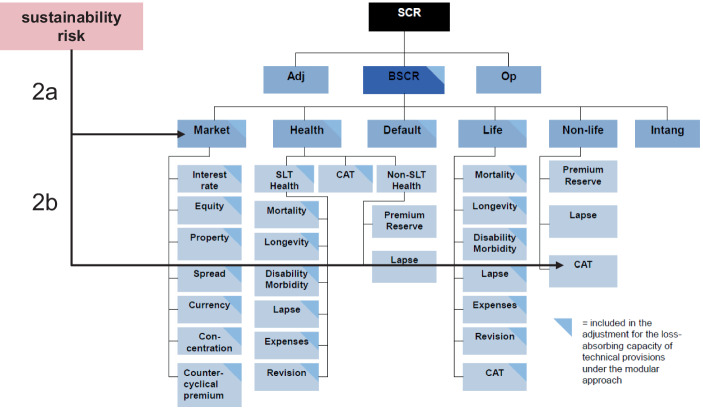

Als weitergehende Alternative mit höherer Plausibilität kann über die Integration von Nachhaltigkeitsrisiken in vorhandene Risikomodule nachgedacht werden (Abb. 4). Naheliegend ist z. B. die Integration von Risiken durch den Klimawandel auf der Aktivseite der Bilanz in das Marktrisikomodul (Variante 2a). Verluste bei Immobilienwerten könnten z. B. auch durch den Klimawandel bedingt sein und höhere Stresse der Marktwerte (direkt) gehaltener Immobilien notwendig machen.

Auf der Passivseite könnten zusätzliche extreme Wetterereignisse (200-Jahres-Ereignis, aber häufiger) modelliert werden und im unternehmensindividuellen Katastrophenrisiko, einem Risikosubmodul im Risikomodul versicherungstechnische Risiken Nicht-Leben berücksichtigt werden (Variante 2b). Im Gegensatz zu den Varianten 1a und 1b, die sich ausschließen, könnten die Varianten 2a und 2b kombiniert werden (evtl. sogar ergänzt werden, um eine Integration von Nachhaltigkeitsrisiken in weitere Risiko(sub)module, vgl. 4.3.3).^59^

Da die Korrelationen sowie in Säule 2 auf den Prüfstand zu stellen sind, könnte eine Neuparametrisierung der Korrelationsfaktoren erfolgen. Falls dies mangels ausreichender Daten nicht mit der erforderlichen Verlässlichkeit erfolgen kann, kann eine konservative Abschätzung die Problematik evtl. auflösen (Korrelationsfaktor = 1).

Auch mit diesen Varianten kann man der Vielfalt und dem zeitlichen Horizont von Nachhaltigkeitsrisiken evtl. nicht gerecht werden (z. B. bzgl. Nachhaltigkeitsrisiken bei Outsourcing), sodass weitergehende Überlegungen notwendig erscheinen.

#### Standardformel: Integration in Risiko(sub)module

Folgende Risikokategorien sieht die BaFin bei beaufsichtigten Unternehmen potenziell betroffen:^60^

- Kreditrisiko/Adressenausfallrisiko
- Markt(preis)risiko
- Liquiditätsrisiko
- Operationelles Risiko
- Versicherungstechnisches Risiko
- Strategisches Risiko
- Reputationsrisiko

**Figure 5:**
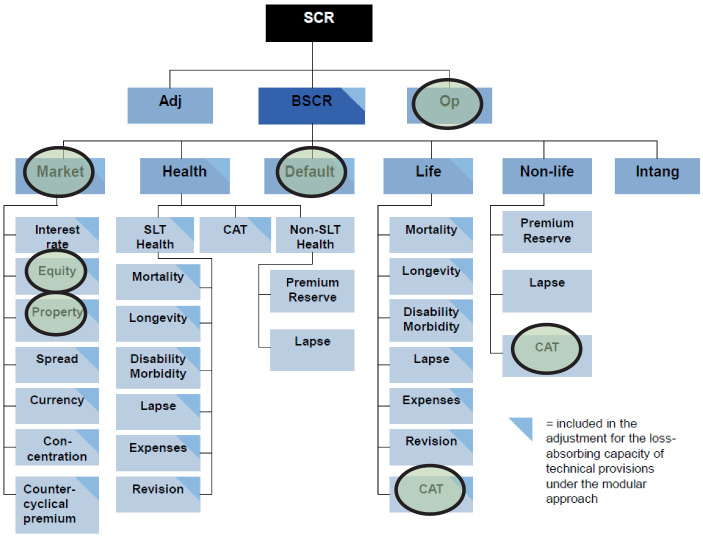

Auch, wenn manche dieser Risiken für Versicherungsunternehmen evtl. nicht wesentlich sind, bleiben bezüglich der Integration in die Säule 1-SCR-Struktur einige Risiken übrig, die nicht explizit quantifiziert werden (vgl. Abb. 5).

Es zeigt sich, dass eine Vielzahl dieser Risikoarten richtigerweise betroffen sind, aber nicht in der SCR-Struktur im Standardansatz (quantitativ) erfasst werden können (z. B. auch nicht in ihrer zeitlichen Struktur wie strategische Risiken auf Ein-Jahres-Sicht). Da der ORSA-Prozess eigentlich keine internen Modelle^61^ und diese auch noch mehrjährig implizieren soll, ergibt sich, dass zusätzliche Betrachtungen durchgeführt werden müssen, um die (zukünftigen) regulatorischen Anforderungen zur Integration relevanter Nachhaltigkeitsrisiken in den ORSA-Prozess und das Risikomanagementsystem zu erfüllen.

## Stress-Tests zu Nachhaltigkeitsrisiken

Stress-Tests werden in der Versicherungspraxis schon lange diskutiert, auch wenn Stress-Tests im Bankenumfeld eine andere (höhere) Bedeutung hatten.^62^ Auch aufsichtliche Stress-Tests für Versicherungen sind schon lange in der Diskussion.^63^ Im Bankenbereich sind nach wie vor beispielsweise Stress-Tests für spezifische Risikokategorien üblich, z. B. zum Kreditrisiko oder zum Marktpreisrisiko. Lange Tradition haben im Versicherungsbereich Stress-Tests für die Kapitalanlagen. Neu ist, dass Stress-Tests (auch) direkt Nachhaltigkeitsrisiken adressieren.

Überraschend ist diese Integration von Nachhaltigkeitsrisiken allerdings nicht, da Szenarioanalysen und Stress-Tests seit jeher zur Beurteilung und Steuerung von Risiken in Unternehmen als auch auf Märkten eingesetzt wurden. Sie sind daher auch regulatorisch im Banken- wie auch in der Versicherungsaufsicht von Aufsichtsbehörden verlangt und um spezifische Aspekte erweitert bzw. angepasst worden (z. B. aufgrund der Finanzkrise 2007/2008 oder zuletzt aufgrund der COVID-19-Pandemie).

Stress-Tests werden zum Teil auf ihre puren Ergebnisse reduziert und manchmal wegen zu hoher oder zu niedriger Wahrscheinlichkeiten für die getesteten Szenarien kritisiert,^64^ sie sind jedoch auch insbesondere als Prozess betrachtet wertvoll.^65^ Dieser Prozess beinhaltet z. B. die Datensammlung als auch die (adressatengerechte) Kommunikation der Stress-Test-Ergebnisse.^66^

Die besondere Herausforderung von Nachhaltigkeits-Stress-Tests besteht in den für Klimawandel-Szenarios zu treffenden Annahmen und deren Unsicherheiten.^67^ Dies gilt in gleicher Weise für Stress-Tests zu Nachhaltigkeitsrisiken, die für mikro- oder makroprudenzielle Zwecke entwickelt werden (Mikro- bzw. Makro-Stress-Tests). Die unterschiedlichen Ziele sind in Tab. 2 aufgeführt.

**Table 2:** 

Ziele Mikro-Stress-Tests	Ziele Makro-Stress-Tests
Bewertung der Resilienz einzelner Versicherungsunternehmen und -gruppen gegenüber den Risiken des Klimawandels und Bewertung des Umfangs potenzieller finanzieller Engagements/Verluste bei adversen Klimaszenarien	Bewertung der Resilienz des gesamten Versicherungssektors und der potenziellen systemischen Risiken des Klimawandels
	Bewertung der potenziellen Auswirkungen von Klimawandelrisiken auf andere Finanzsektoren und die Realwirtschaft
Verbesserung des Verständnisses der potenziell langfristigen Auswirkungen des Klimawandels auf bestehende Geschäftsmodelle und mögliche chancenreiche neue Geschäftsmodelle (InsurTechs)	Bewertung der potenziellen Auswirkungen auf die künftige Versicherbarkeit von Risiken und der potenziellen Schutzlücke („protection gap“) für die Realwirtschaft durch Klimawandel
Verbesserung des Risikomanagements zur Bewertung und Transfers der Risiken des Klimawandels	Abschätzung der notwendigen Präventionsmaßnahmen (z. B. hinsichtlich Hochwasserschutz und Gesundheitsförderung)

^a^Quelle: in Anlehnung an EIOPA (2022, S. 10)

Um diese Ziele zu erreichen, werden im Folgenden Ausgestaltungsempfehlungen im Sinne von Referenz-Punkten gegeben. Unbenommen bleibt, dass damit keine vollständigen Lösungen erreicht werden, sondern nur eine Referenz-Architektur von Strukturen und die konkrete Umsetzung (unternehmensindividuell oder marktbezogen) herausfordernd bleibt.

### Mikro-Stress-Tests zu Nachhaltigkeitsrisiken

Für die Ausgestaltung von Mikro-Stress-Tests werden folgende Referenz-Punkte vorgeschlagen, die eine Referenz-Architektur von Stress-Tests zu Nachhaltigkeitsrisiken für (einzelne) Versicherungsunternehmen und -gruppen darstellen können:^68^

- In Mikro-Stress-Tests sollten physische und transitorische Risiken (idealerweise inkl. Haftungsrisiken) und die Zusammenhänge zwischen ihnen betrachtet werden.
- Die Auswirkungen von Nachhaltigkeitsrisiken sollten sowohl aktiv- als auch passivseitig berücksichtigt werden.
- Es sollten mehrere Transitionspfade einbezogen werden, um die unterschiedlichen Kombinationen des Trade-offs zwischen Transitionsrisiken und den damit verbundenen (hoffentlich vermiedenen) physischen Risiken zu erfassen.
- Bezüglich des Zeithorizonts sollten neben kurz- und mittelfristigen Szenarien auch langfristige Betrachtungen, ggfs. eher qualitativer Natur, angestellt werden.
- Akute und chronische Risiken des Klimawandels sollten in den Stress-Tests vorgesehen sein, um sowohl die kurzfristigen Effekte als auch die langfristigen Auswirkungen zu erfassen.

Zu diesen Referenz-Punkten werden nun noch ausgewählte Aspekte ergänzt bzw. konkretisiert:

- Für die Ausgestaltung geeigneter Szenarien kann insbesondere auf die Vorarbeiten des NGFS zurückgegriffen werden, die vier Referenz-Felder für Szenarien über die zwei Dimensionen physische Risiken und transitorische Risiken definieren (Referenz-Matrix).^69^
- Als Referenz-Szenarios können die im 6. IPCC-Bericht (IPCC AR6) entwickelten herangezogen werden (sog. Shared Socioeconomic Pathways, SSPs).
- Hinsichtlich der Granularität der Betrachtungen sollte der Grundsatz der Proportionalität angewendet werden, der bei Mikro-Stress-Tests eine detaillierte Betrachtung an den Stellen erfordert, bei denen das Risikoprofil gegenüber Nachhaltigkeitsrisiken anfällig und mit erheblichen Auswirkungen verbunden sein kann:^70^
  - Bezüglich der Kapitalanlagen sollte mindestens nach Anlageklassen, Sektoren und Ländern unterschieden werden.
  - Beim Versicherungsgeschäft bieten sich Unterscheidungen nach Versicherungszweigen oder zumindest nach den Sparten Leben, Kranken und Nicht-Leben an.
  - Umgekehrt kann an anderen Stellen eine zunächst nur pauschalere Betrachtung sinnvoll sein.
- Klimawandel-Stress-Testing erfordert wegen der allmählichen Realisierung des Risikos über einen langen Zeitraum andere Zeithorizonte in der Betrachtung.^71^ Zudem sind die Veränderungen teilweise bereits realisiert und Auswirkungen bereits heute spürbar. Vor dem Hintergrund sind standardisierte Methoden bisher nicht gefunden und auch die Entwicklung ist als sehr dynamisch anzusehen.^72^ Lt. BaFin haben deutsche Versicherungsunternehmen allerdings hinsichtlich der ESG-Kriterien noch keine Ausweitung des Planungshorizonts (von üblicherweise 3–5 Jahren) vorgenommen.^73^ Es is darüber nachzudenken, für einzelne Entscheidungsfelder den Betrachtungszeitraum auszudehnen und damit differenzierte Horizonte für z. B. neue Geschäftsfelder zu setzen.
- Für langfristige Betrachtungen sind weniger rein quantitative Berechnungen zielführend, sondern auch qualitative Abschätzungen, sodass eine Kombination quantitativer und qualitativer Elemente entscheidungsnützlich sein könnte, z. B. hinsichtlich strategischer Risiken für spezifische Geschäftsmodelle.
- Die Modellierungsansätze sind je nach Risikoart jeweils in Bezug auf transitorische oder physische Risiken spezifisch zu wählen (vgl. 4.3).^74^ Zur Kalibrierung sind möglichst historische Daten, aber auch hypothetische Daten einzubeziehen.

Die Dynamik der Entwicklung geeigneter Modelle, die auch frei/öffentlich verfügbar sind, ist im vollen Gange. Für kleinere und mittlere Unternehmen ist der Weg, eigener Modellierungen nur bedingt gangbar, sodass z. B. auch Versicherungsverbänden eine wichtige Aufgabe zukommt, gemeinsam entwickelte Lösungen bereitzustellen.

### Makro-Stress-Tests zu Nachhaltigkeitsrisiken

Als Referenz-Eckpunkte können die für Mikro-Stress-Tests skizzierten von Aufsichtsbehörden adaptiert werden. Im Fokus wird nun der EU-Versicherungsmarkt stehen, für die die regulatorischen Entwicklungen hinsichtlich Nachhaltigkeitsrisiken skizziert wurden, insbesondere hinsichtlich ihrer Einbeziehung in Säule 2 und auch Säule 1 von Solvency II (vgl. 3.2).

2021 führte EIOPA bereits den 5. Versicherungs-Stress-Test durch.^75^ Für die Zukunft ist vorgesehen, auch verstärkt Nachhaltigkeitsrisiken in dem Stress-Test mitabzutesten. Das Mandat dafür hat die Europäische Kommission schon vorgesehen.

EIOPA hat Ende Januar 2022 ein drittes Papier in einer Reihe von Papieren zu den methodischen Grundsätzen zu aufsichtlichen Stress Tests im Versicherungssektor veröffentlicht.^76^ Das methodische Papier konzentriert sich auf die Klimawandelkomponente und stellt einen weiteren Schritt zur Ausgestaltung eines EIOPA-Stress-Test-Rahmens dar.

Insbesondere werden in dem Papier methodische Grundsätze dargelegt, die für die Gestaltung von Bottom-up-Stress-Tests verwendet werden können, die darauf abzielen, die Anfälligkeit von Versicherungsunternehmen und -gruppen gegenüber Klimarisiken zu bewerten. Obwohl das Auftreten von Klimarisiken nicht unerwartet ist, sieht EIOPA diese im Vergleich zu anderen versicherungsspezifischen und finanziellen Risiken als relativ neu an.^77^ Zu begrüßen ist, dass die Einbeziehung dieser Risiken nun auch zu einer Priorität für politische Entscheidungsträger und Aufsichtsbehörden gleichermaßen geworden ist.^78^

Jeder Klimawandel-Stress-Test sollte lt. EIOPA zum jetzigen Zeitpunkt als Teil einer Lernkurve für die Branche und die Aufsichtsbehörden betrachtet werden, die sich in Zukunft sicherlich weiterentwickeln wird. Schon jetzt sind – gemäß EIOPA – Klima-Stress-Tests ein wichtiges Instrument, um:

- das Bewusstsein für klimabezogene Risiken zu schärfen,
- zu verstehen, wie Versicherer solche Risiken einschätzen,
- die Fähigkeiten des Risikomanagements zu verbessern sowie
- potenzielle Spillover-Effekte auf andere Teile des Finanzsektors und auf die Realwirtschaft zu bewerten.^79^

In Anbetracht der zunehmenden Berücksichtigung von Klimarisiken durch die Versicherungsbranche und die Aufsichtsbehörden auf europäischer und globaler Ebene und mangels eines allgemeinen Rahmens für Klima-Stress-Tests für den Versicherungssektor in der EU werden in diesem Papier konzeptionelle Ansätze für die Bewertung von Klimarisiken für Versicherer unter ungünstigen Szenarien vorgestellt. Diese könnten auch für die nationalen Aufsichtsbehörden maßgeblich sein, um zu vermeiden, dass jeweils unterschiedliche Ansätze in den einzelnen EU-Mitgliedstaaten gewählt werden, sodass die Auswirkungen auf die nationalen Finanzmärkte nicht oder nur sehr bedingt verglichen bzw. zu Betrachtungen des EU-Versicherungsmarktes aggregiert werden können.

Da zumindest kurzfristig nur bedingt auf die spezifischen Mikro-Stress-Tests der Versicherungsunternehmen und -gruppen zurückgegriffen werden kann (weil noch in Entwicklung), ist der Weg über standardisierte Vorgaben für den Teilnehmerkreis der bisherigen Versicherungs-Stress-Tests gangbar (ca. 30 bis 40 große, internationale Versicherungsgruppen, nach Größe bzw. nationaler Bedeutung ausgewählt).

**Figure 6:**
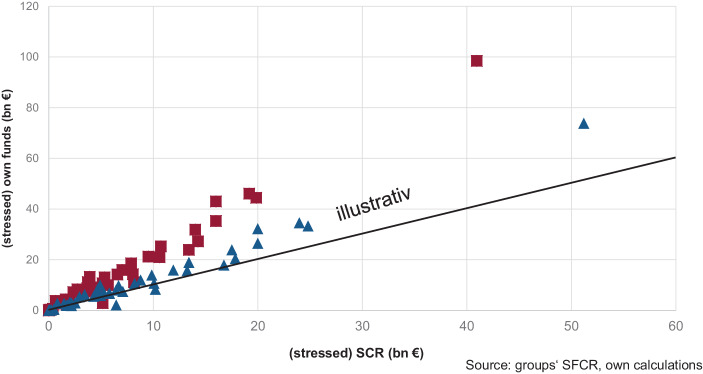

Zwischen der Solo-Betrachtung und der Gesamtmarktbetrachtung im Hinblick auf Nachhaltigkeitsrisiken könnte – wie allgemein auch – eine „Gruppenbetrachtung“ der Auswirkungen des Klimawandels wertvoll sein. In Abb. 6 ist eine solche illustrativ dargestellt. Sie zeigt das Verhältnis der Eigenmittelhöhe zum (Gruppen‑)SCR vor Stress (viereckig, echte Daten) bzw. nach Stress (dreieckig, illustrative Daten) für die Unternehmen, die am EIOPA-Versicherungs-Stress-Tests 2020 teilgenommen haben (sofern die Daten verfügbar waren). Der Stress für die Eigenmittel ist simuliert über einen Anteil „grüner“ Kapitalanlagen mit geringerem Stress und einem höheren Stress für die (z. B. nicht-Taxonomie-konformen) restlichen Kapitalanlagen. Das gestresste SCR ergibt sich aus einer pauschalen Annahme zur Erhöhung physischer Risiken mit einem höheren versicherungstechnischen Risiko. Während vor dem Stress die Punkte größtenteils einen großen Abstand zur eingezeichnete Ursprungsgerade haben, die einer Solvenzquote von 100 % entspricht, nähern sich die (dreieckigen) Punkte dieser Linie an bzw. unterschreiten sie sogar in einzelnen Fällen, sodass sich ein (illustratives) Bild der Finanzstabilität eines großen Teils des EU-Versicherungsmarktes ergibt.

Die Gefahr standardisierter Stress-Tests ist, dass relevante Organisationseinheiten innerhalb der Versicherungsunternehmen und -gruppen nicht ausreichend in das Stress-Testing eingebunden sind (evtl. sogar eine Beauftragung externer Beratungsunternehmen erfolgt), sodass u. a. wegen geringerer Akzeptanz der Stress-Tests auch deren Ergebnisse unter Umständen nicht ausreichend in die Entscheidungsprozesse einbezogen werden.^80^

Aufgabe der „gestressten“ Versicherungsunternehmen und -gruppen ist auch eine adressengerechte Kommunikation der Ergebnisse an die Stakeholder, beispielsweise ist die Erwartungshaltung der Öffentlichkeit bezüglich der Absicherung von Naturkatastrophen (über z. B. eine Versicherungspflicht bei der Gebäudeversicherung zu einer zusätzlichen Elementarschadenversicherung) groß.

Dringend notwendig wäre beispielsweise auch, zu testen, ob durch Naturkatastrohen bei zunehmenden Klimawandel zukünftig Teile des Versicherungsmarktes ausfallen und ggfs. Insolvenzsicherungssysteme greifen müssten, um die Schäden für die Versicherten zu zahlen. In solchen Makro-Stress-Tests ist zu überlegen, ob Rückversicherungsschutz als vollständig risikomindernd angesehen wird oder ob auch der Rückversicherungsschutz zumindest teilweise ausfallen könnte, was dann im Weiteren die Frage staatlicher Haftung aufwirft.

## Zusammenfassung und Ausblick

Die Klimakrise wird weiterhin die politische Agenda prägen. Insbesondere wird die Fridays for Future-Bewegung keine temporäre Erscheinung bleiben, sondern in vielen Ländern weiter einen großen Einfluss auf die Öffentlichkeit haben. Auch, wenn sich darin besonders junge Menschen engagiert und für das Bewusstsein des Klimawandels demonstriert haben, werden Nachhaltigkeitsziele von der breiten Bevölkerungsmehrheit in der EU getragen. Auch, wenn die Anzahl der Demonstrationen oder die Anzahl der Teilnehmenden in Zukunft abnehmen sollte, wird der Trend zur Nachhaltigkeit Regierungen weiterhin darin erinnern, die völkerrechtlich eingegangenen Verpflichtungen zum Klimaschutz auch konkret umzusetzen.

Unternehmen tun daher im Einklang mit diesen politischen Rahmenbedingungen gut daran, ihre Kohleinvestitionen zu reduzieren. Dies kann auch als ein Grund für den Rückzug von Versicherungsunternehmen als Investoren gesehen werden. Viele Versicherungsunternehmen haben sich wie auch die deutsche Versicherungsbranche insgesamt als institutionelle Anleger mit langfristigen Anlagehorizont bereits abgewendet; dies wird sich auch in Richtung einer Abkehr von der Versicherung von beispielsweise Kohlekraftwerken oder -minen fortsetzen. Die Versicherungsbranche wird sich auch darüber hinaus aktiv an diesem Wandel beteiligen, indem sie Nachhaltigkeitsstrategien als Teil ihrer Geschäftsstrategien, insbesondere auch ihrer daraus abgeleiteten Risikostrategien, entwickelt.

Als Basis für diese Veränderung sind Nachhaltigkeitsrisiken zunächst zu identifizieren, dann zu bewerten und quantifizieren, damit die entsprechenden Risikomaßnahmen getroffen und überwacht werden können. Für die Identifikation der Nachhaltigkeitsrisiken sind traditionelle Ansätze der Risikomessung nicht ausreichend, insbesondere weil historische Datenreihen über z. B. zunehmende physische Schäden nicht vorliegen (können) oder die Abschätzung von transitorischen Risiken einer hohen Unsicherheit wegen der Diskrepanz zwischen politischen Zielen und tatsächlichen Maßnahmen unterliegt.

Es ist daher hochgradig sinnvoll, Nachhaltigkeitsrisiken durch Szenarioanalysen und Stress-Tests zu adressieren. Die regulatorischen Entwicklungen zu Nachhaltigkeitsrisiken auf globaler, europäischer Ebene und nationaler Ebene laufen darauf hinaus. Speziell die EU-Vorgaben sehen zunehmend auch Quantifizierungen rechtlich bindend (!) vor. Aufsichtsbehörden haben Nachhaltigkeit zu ihren strategischen Prioritäten erhoben und werden – wie die BaFin über eher unkonventionelle Methoden wie „Merkblätter“ und Umfragen – zusätzlich zur Gesetzgebung und Rechtssetzung Druck entfalten.^81^

Bei der Ausgestaltung dieser Szenarioanalysen und Stress-Tests zu Nachhaltigkeitsrisiken sind verschiedene grundlegende Annahmen zu treffen, z. B. über die betrachteten Zeithorizonte und die Wahrscheinlichkeiten für z. B. Extremwetterereignisse und CO_2_-Preiserhöhungen. Dass diese Annahmen evtl. nicht Realität werden, spricht nicht dagegen, Szenarioanalysen und Stress-Tests durchzuführen. Es liegt vielmehr in der Natur der Sache und der Instrumente, mit Unsicherheit umzugehen. Kein Risikomanagement ist keine Alternative.

Da die Risiken unternehmensindividuell als auch marktweit auftreten können, sind Stress-Tests für mikro- und makroprudenzielle Zwecke erforderlich. Die Ausgestaltung von Klimawandel-bezogenen Stress-Tests ist spezifisch, aber die Logik und die Ziele sind wie auch bei „normalen“ Stress-Tests. Aufgrund der langfristigen Natur der Risiken aus dem Klimawandel ergibt sich für Stress-Tests auch die Notwendigkeit eines stärker explorativen Charakters im Vergleich zu einem traditionellen Stress-Tests, der vornehmlich finanzielle Risiken adressiert.^82^

Für die Mikro- und Makro-Stress-Tests wurden Referenz-Eckpunkte dargestellt, die Referenz-Architekturen für Stress-Tests ergeben. Deren konkrete Ausgestaltung wird Unternehmen bzw. Aufsichtsbehörden überlassen bleiben, insbesondere hinsichtlich Granularität und Standardisierung.

In Anbetracht der Veränderungen durch die Klimakrise ist klar, dass auch die Versicherungsregulierung „grün“ werden muss. Dazu ist es notwendig, den Erfolg von Nachhaltigkeitsstrategien zu gewährleisten. Dies erfordert nicht zuletzt ein engagiertes Change Management bei allen Stakeholdern. Die Versicherungsunternehmen müssen gemeinsam mit ihren Kunden an ihren Prozessen, Kontrollmechanismen, Messkriterien und Anreizen arbeiten. Das Gleiche gilt für Aufsichtsbehörden und ihr Personal. Die wissenschaftlichen Erkenntnisse der Klimaforschung sollten dabei weiterhin Ausgangspunkt bleiben und auch möglichst interdisziplinär in der versicherungsmathematischen, versicherungsökonomischen bzw. versicherungsrechtlichen Forschung noch stärker Eingang finden.

## Anhang

**Table 3:**

Thema	Kriterium	Beschreibung	Ausschluss
Landminen	Antipersonenlandminen, Hersteller von Waffensystemen	Unternehmen, die Antipersonenlandminen für Waffensysteme herstellen. Umfasst Waffen oder Munition, die ähnlich funktionieren oder dieselbe Funktion erfüllen wie traditionelle Antipersonenminen	Jeglicher Verstoß
Arbeitsrechte	Kinderarbeitsflagge	Dieser Indikator misst die Schwere von Kinderarbeitsstreitigkeiten. Zu den Faktoren, die sich auf diese Bewertung auswirken, gehören unter anderem eine Vorgeschichte der Beteiligung an Rechtsstreitigkeiten im Zusammenhang mit Kinderarbeit, weit verbreitete oder ungeheuerliche Fälle von Kinderarbeit, Widerstand gegen verbesserte Praktiken und Kritik von NGOs und/oder anderen externen Beobachtern	Rote Flagge
Glücksspiel	Maximaler Prozentsatz des Umsatzes	Der Prozentsatz des Umsatzes des letzten Jahres oder maximal geschätzter Prozentsatz, den ein Unternehmen aus geschäftsbezogenen Aktivitäten in Zusammenhang mit Glücksspiel ableitet	Größer 10 %
Waffen	Nukleare Systeme	Unternehmen, die Atomwaffen herstellen, einschließlich nuklearer Sprengköpfe, ballistischer Interkontinentalraketen und U‑Boote ballistischer Raketen, die nukleare Sprengköpfe liefern können	Alle
Unterhaltung für Erwachsene	Maximaler Prozentsatz der Einnahmen	Der Prozentsatz des Umsatzes des Jahres (oder maximal geschätzter Prozentsatz), den ein Unternehmen von der Erwachsenenunterhaltung erhalten hat	Größer 30 %
Streumunition	Hersteller von Waffensystemen	Unternehmen, die Waffensysteme für Streumunition herstellen	Größer 30 %
Tabakkonsum	Maximaler Prozentsatz des Umsatzes	Der Prozentsatz des Umsatzes des letzten Jahres oder maximal geschätzter Prozentsatz, den ein Unternehmen aus geschäftlichen Aktivitäten im Zusammenhang mit Tabak gewonnen hat	Größer 10 %
Thermische Kohle	Maximaler Prozentsatz des Umsatzes	Dieser Faktor gibt den maximalen Prozentsatz des Umsatzes (entweder angegeben oder geschätzt) von mehr als 0 % an, den ein Unternehmen durch den Abbau von Steinkohle (einschließlich Braunkohle, Steinkohle, Anthrazit und Kraftwerkskohle) und seinen Verkauf an Dritte erzielt. Davon ausgenommen sind: Einnahmen aus metallurgischer Kohle; Kohle, die zur internen Stromerzeugung gefördert wird (z. B. bei vertikal integrierten Stromerzeugern), innerbetrieblicher Verkauf von gewonnener thermischer Kohle und Einnahmen aus dem Kohlehandel	Größer 30 %
Erzeugung thermischer Kohle	Maximaler Prozentsatz des Umsatzes	Dieser Faktor gibt den maximalen Proentsatz des Umsatzes (entweder gemeldet oder geschätzt) an, den ein Unternehmen aus der auf Kohlekraftwerken basierenden Stromerzeugung erzielt	Größer 30 %
CO_2_-Emissionen	Scope 1 + 2 Intensität (t/€ Mio. Umsatz)	Diese Zahl stellt die zuletzt gemeldeten oder geschätzten Scope 1 + Scope 2-Treibhausgasemissionen des Unternehmens dar, die durch den Umsatz in Euro normalisiert wurden, was einen Vergleich zwischen Unternehmen unterschiedlicher Größe ermöglicht	Unternehmen im schlechtesten Viertel der Unterbranche und schlechter als Unterbranchenschnitt und Unterbranchenschnitt schlechter als Durchschnitt des MSCI World
Alkohol	Maximaler Prozentsatz des Umsatzes	Der Prozentsatz des Umsatzes des letzten Jahres oder maximal geschätzter Prozentsatz, den ein Unternehmen aus der Herstellung, dem Vertrieb, dem Einzelhandel, der Lizenzierung und der Lieferung alkoholischer Produkte gewonnen hat	Größer 10 %
Stammzellforschung	Embryonal	Unternehmen, die Stammzellforschung mit aus menschlichen Embryonen gewonnenen Zellen betreiben	Alle
Gentechnik	Landwirtschaft	Unternehmen, die Pflanzen wie Saatgut und Getreide sowie andere Organismen, die für die landwirtschaftliche Zwecke oder dem menschlichen Verzehr bestimmt sind, genetisch verändern. In diese Kategorie fallen auch Unternehmen, die USDA APHIS-Genehmigungen für Feldtests, Meldungen oder den Deregulierungsstatus von gentechnisch veränderten Pflanzen beantragt haben	Alle
Waffen	Maximaler Prozentsatz des Umsatzes	Der Prozentsatz des Umsatzes des letzten Jahres (oder maximal geschätzter Prozentsatz), den ein Unternehmen aus Waffensystemen, Komponenten sowie Unterstützungssystemen und -diensten gewonnen hat	Größer 10 %
Ölsand	Maximaler Prozentsatz des Umsatzes	Dieser Faktor gibt den maximalen Prozentsatz des Umsatzes (entweder gemeldet oder geschätzt) von mehr als 0 % an, den ein Unternehmen aus der Ölsandförderung für eine Reihe von Unternehmen zieht, die Ölsandreserven besitzen und Nachweise für die Gewinnung von Erträgen aus der Ölsandgewinnung offenlegen. Dieser Faktor beinhaltet keine Einnahmen aus Nicht-Extraktionsaktivitäten (z. B. Exploration, Vermessung, Verarbeitung, Raffination). Besitz von Ölsandreserven ohne damit verbundene Gewinnerlöse, Einnahmen aus innerbetrieblichen Verkäufen	Größer 0 %
Schieferöl	Maximaler Prozentsatz des Umsatzes	Dieser Faktor gibt den maximalen Prozentsatz des Umsatzes (entweder gemeldet oder geschätzt) von mehr als 0 % an, den ein Unternehmen aus der Schieferölproduktion erzielt. Dieser Faktor beinhaltet keine Einnahmen aus Nicht-Extraktionsaktivitäten (z. B. Exploration, Vermessung, Verarbeitung, Raffination); Besitz von Schiefergasreserven ohne damit verbundene Gewinnerlöse, Einnahmen aus innerbetrieblichen Verkäufen	Größer 0 %
Tierschutz	Nichtmedizinische Tests	Unternehmen, die Tierversuche für nichtpharmazeutische Produkte wie Kosmetika, Körperpflegeprodukte und Haushaltsreinigungsprodukte durchführen	Alle

## Footnotes

1 Zum Einstieg in die wissenschaftlichen Grundlagen des Klimawandels z. B. Rahmstorf und Schellnhuber (2019).

2 Vereinte Nationen (2015a).

3 Vereinte Nationen (2015b).

4 Zu Klimapolitik siehe z. B. Edenhofer und Jakob (2019).

5 Europäische Kommission (2019b).

6 Vgl. z. B. Gatzert et al. (2020, S. 313).

7 Vgl. NGFS (2019a).

8 Europäische Kommission (2019a, S. 1).

9 Risiken können zweiseitig definiert werden, sodass – als Gegenstück von Nachhaltigkeits*risiken* – auch Nachhaltigkeits*chancen* betrachtet werden. Der Fokus liegt hier aber stärker auf negativen Abweichungen. Für Chancen aus der Nachhaltigkeit für Versicherer siehe beispielsweise Gatzert et al. (2020, S. 315 ff.).

10 Zum Nachhaltigkeits-Controlling bei Versicherern vgl. Kraft (2019).

11 BaFin (2019, S. 11).

12 Vgl. Scherenberg (2011, S. 131).

13 Vgl. Scherenberg (2011, S. 131).

14 Vgl. Munich Re (2020).

15 Vgl. Munich Re (2020).

9999 Vgl. Allianz (2018).

16 Für eine grobe Gegenüberstellung für ausgewählte große Versicherungskonzerne siehe Kraft (2019, S. 77).

17 So z. B. Talanx vgl. Talanx (2019).

18 Mehr Informationen unter https://www.unepfi.org/net-zero-alliance/about/ [05.02.2022].

19 Zu beispielhaften Aufsichtspraktiken auch in anderen Ländern siehe z. B. IAIS (2021).

20 Siehe z. B. TCFD (2020).

21 Vereinte Nationen (2015b).

22 Vgl. dazu und im Folgenden Vereinte Nationen (2015b).

23 Vgl. Vereinte Nationen (2015a).

24 Vereinte Nationen (2015a).

25 Vgl. Europäische Kommission (2018).

26 Vgl. dazu und im Folgenden EIOPA (2019b).

27 EIOPA (2019a).

28 EIOPA (2019a).

29 Vgl. dazu und im Folgenden Europäische Kommission (2021a).

30 Vgl. dazu EIOPA (2019b, u. a. S. 10 bzw. 19). Vorangegangen war eine öffentliche Konsultation der Stakeholder (EIOPA 2018) und eine formelle Anfrage (Europäischen Kommission 2018).

31 Vgl. dazu und im Folgenden EIOPA (2021d).

32 Vgl. dazu und im Folgenden Europäische Kommission (2021b).

33 Eine solche Integration von Nachhaltigkeitsrisiken im Risikomanagement nach Solvency II hatte die Europäische Kommission angekündigt. Vgl. dazu Europäische Kommission (2021c, S. 14 („Action 3“)).

34 Vgl. EIOPA (2021e).

35 Vgl. dazu EIOPA (2021c).

36 Fallbeispiele und Aufsichtspraktiken aus diversen Staaten finden sich beispielsweise in IAIS (2021).

37 BaFin (2019).

38 Vgl. BaFin (2019, S. 2–5).

39 Vgl. BaFin (2018).

40 Vgl. BaFin (2021a).

41 BaFin (2022).

42 BaFin (2022).

43 BaFin (2022).

44 Vgl. BaFin (2022).

45 Vgl. EIOPA (2021a).

46 BaFin (2021a). Siehe auch BaFin (2019, S. 11 f.), EIOPA (2021a, S. 6 f.), The Geneva Association (2021, S. 13), EIOPA (2022, S. 6).

47 BaFin (2021a, S. 14). Vgl. auch BaFin (2019, S. 11 f.), EIOPA (2021a, S. 6), The Geneva Association (2021, S. 13), EIOPA (2022, S. 6).

48 Vgl. BaFin (2019, S. 11 f.).

49 Vgl. BaFin (2019, S. 13).

50 Zu Ausschlusskriterien vgl. auch BaFin (2019, S. 23 f.).

51 Vgl. BaFin (2021a, S. 14).

52 Vgl. BaFin (2021a, S. 14).

53 Vgl. BaFin (2021a, S. 14).

54 Siehe https://www.ngfs.net/ngfs-scenarios-portal/ [05.02.2022].

55 Vgl. BaFin (2021a, S. 22).

56 Das Risiko für immaterielle Vermögensgegenstände SCR_intangibles_ wird hingegen nur hinzuaddiert.

57 BSCR bezeichnet das Basis-SCR, das durch Anpassungen für die zukünftige Überschussbeteiligung und latente Steuern sowie dem SCR für operationelle Risiken zum SCR führt.

58 Auch die BaFin lehnt eine separate Risikoart „Nachhaltigkeitsrisiken“ wegen Abgrenzungsschwierigkeiten ab. Vgl. BaFin (2019, S. 14).

59 Zu bedenken ist lt. BaFin auch, dass Versicherungsunternehmen durch dasselbe Nachhaltigkeitsrisiko sowohl aktiv- wie passivseitig getroffen werden können. Vgl. BaFin (2019, S. 14).

60 BaFin (2019, S. 14).

61 Vgl. Erwägungsgrund 36 SII-RL (2009).

62 Vgl. z. B. Klauck (2006), Rusnak (2010).

63 Vgl. z. B. Bennemann und Schalk (2010).

64 Vgl. z. B. Europäischer Rechnungshof (2018).

65 Zum Stress-Test-Prozess vgl. z. B. EIOPA (2020, S. 9 f.).

66 Gerade im Hinblick auf Nachhaltigkeitsrisiken ist es wichtig, dass Stakeholder die Ergebnisse von Stress-Tests verstehen und einordnen können. Vgl. The Geneva Association (2021, S. 29).

67 Siehe dazu z. B. Table 1‑1 (Key assumptions and uncertainties surrounding climate change scenarios) in EIOPA (2022, S. 4 f.).

68 Vgl. EIOPA (2022, S. 11).

69 Vgl. EIOPA (2022, S. 12). Siehe vor allem NGFS (2019a), NGFS (2019b), NGFS (2020) und NGFS (2021).

70 Vgl. zu einer vertieften Diskussion der Granularität beispielsweise EIOPA (2022, S. 14).

71 Vgl. EIOPA (2022, S. 4).

72 Vgl. EIOPA (2022, S. 4).

73 Vgl. BaFin (2021a, S. 15).

74 Ausführlich dazu z. B. EIOPA (2022, S. 18-36).

75 Vgl. EIOPA (2021e). Zu einer Übersicht aufsichtlicher Klima-Stress-Tests siehe EIOPA (2022, S. 42–46).

76 EIOPA (2022).

77 Vgl. EIOPA (2022, S. 4).

78 Vgl. EIOPA (2022, S. 4).

79 Vgl. EIOPA (2022, S. 5).

80 Vgl. Fremdt und Völz (2010, S. 8).

81 Die BaFin hat Nachhaltigkeit in ihren 10 Mittelfristzielen aufgenommen. Vgl. BaFin (2021b, S. 3).

82 Vgl. EIOPA (2022, S. 9).
